# Silencing of *SOD1* sensitises *ATRX*-deficient cells to camptothecin treatment through increased activity of the alternative lengthening of telomeres pathway

**DOI:** 10.1093/hmg/ddaf118

**Published:** 2025-08-01

**Authors:** Natalie Mattis, Tomas Goncalves, Kanggeon Kim, Ester M Hammond, Anna M Rose

**Affiliations:** Department of Paediatrics, University of Oxford, John Radcliffe Hospital, Headley Way, Oxford, OX3 9DU, United Kingdom; Department of Biomedical Sciences, Oxford Brookes University, Gipsy Lane, Oxford, OX3 0BP, United Kingdom; Department of Paediatrics, University of Oxford, John Radcliffe Hospital, Headley Way, Oxford, OX3 9DU, United Kingdom; MRC Weatherall Institute of Molecule Medicine, University of Oxford, Headley Way, Oxford, OX3 9DU, United Kingdom; Department of Oncology, University of Oxford, ORCRB, Roosevelt Drive, Oxford, OX3 7DQ, United Kingdom; Department of Oncology, University of Oxford, ORCRB, Roosevelt Drive, Oxford, OX3 7DQ, United Kingdom; Department of Paediatrics, University of Oxford, John Radcliffe Hospital, Headley Way, Oxford, OX3 9DU, United Kingdom; MRC Weatherall Institute of Molecule Medicine, University of Oxford, Headley Way, Oxford, OX3 9DU, United Kingdom

**Keywords:** telomere, atrx, dna-protein crosslinks, alternative, lengthening of telomeres

## Abstract

The alternative lengthening of telomeres (ALT) pathway is a telomere maintenance mechanism that is driven by formation of DNA double-strand breaks at telomeres. ALT-positive malignancies often have mutational deletion of *ATRX*, but formation of DNA-protein complexes (DPCs) and elevated reactive oxygen species (ROS) also play a role in the induction of the ALT pathway. It has been recognised that excessive ALT activation can lead to rapid cell death, due to genome instability. Our objectives were to assess whether combining ROS-forming and DPC-forming treatments had a synergistic effect in ATRX-deficient cells. We found that *SOD1* silencing was an effective method for inducing cell death in ATRX-deficient osteosarcoma cell lines; further, this approach was more effective in ATRX-null HeLa-LT than ATRX-wildtype cells. We also observed that dual treatment with DPC-forming chemotherapy (camptothecin) and SOD1 silencing led to a significantly higher level of DPCs, as well as signs of ALT pathway overactivity. Finally, our investigation demonstrated that pre-treatment of ATRX-null cells with shSOD1 significantly increased cellular sensitivity to camptothecin, with synergy between the two treatments. This research provides critical understanding to inform new treatment approaches—which might eventually improve survival for affected individuals, and reduce long-term effects, for survivors of ALT-positive malignancies.

## Introduction

The storage of DNA in linear chromosomes poses a problem for DNA replication, as polymerase enzymes are unable to copy the distal ends, therefore leading to the gradual shortening of the genetic sequence with each cell division. To combat catastrophic loss of genetic information, the genome has developed specialised nucleoprotein structures, telomeres. Under most circumstances, once telomeres reach a critically short length, the cell will enter cellular *senescence* or crisis. Telomere crisis can lead to cell death by apoptosis. By contrast, cancerous cells evolve a telomere maintenance mechanism (TMM), which allows cells to divide without limit and avoid cellular crisis. The majority of human malignant cells re-activate telomerase, an enzyme which replenishes telomeres by adding tandem repeat sequence to chromosome ends. However, up to 20% of all cancer types use a second type of TMM, called alternative lengthening of telomeres (ALT) [1]. ALT pathway activity is most frequently seen in cancers of mesenchymal origin (such as osteosarcoma, soft tissue sarcoma) and central nervous system or neural crest malignancies (such as glioblastoma and neuroblastoma) and, as such, is particularly relevant to cancers affecting children, teenagers and young adults [1, 2]. ALT-positive cancers often display aggressive clinical phenotypes, which results in a poor prognosis when compared to non-ALT equivalent cancers [3–5].

ALT telomere elongation is a specialised form of break-induced replication (BIR) that occurs in the G2 and M phases of the cell cycle and depends on the formation of DNA-double strand breaks (DSBs) [6–9]. Mutational deletion of the *ATRX* gene is a frequent characteristic of ALT-positive malignancies. ATRX protein has multiple roles in genome stability and maintenance; for example, it is involved in the resolution of non-canonical DNA secondary structures (such as G4s and R-loops), prevention and re-start of replication fork stalling, and repair of DSBs (reviewed in [10]). Despite these well-defined roles in genome structure and maintenance, mutational loss of this gene is necessary *but not sufficient* to induce the ALT pathway in cellular systems [11]. Our recent research has proposed other factors which might be important in induction of the ALT-pathway in cells lacking ATRX: formation of DNA-protein complexes (DPCs) and elevated levels of reactive oxygen species (ROS) [12, 13].

DPCs form when DNA-associated proteins—such as TOP1, TOP2 or PARP1—become tightly bound to the DNA strand, either covalently or by other means. We recently observed that treatment of ATRX-deficient cells with DPC-forming drugs led to an induction of ALT-pathway activity [12]. Further, natural ALT-positive cell lines were found to harbour higher levels of DPCs (such as TOP1 covalent complexes (TOP1ccs)) than ALT-negative cell lines [12]. In addition, ATRX*-*deficient cancers have been found to be sensitive to chemotherapeutic agents that form TOP1 or poly-ADP ribose polymerase (PARP) DPCs, such as irinotecan or olaparib [14]. This suggests that modulation of DPCs might be a viable therapeutic strategy for ALT-positive cancer cells. In DNA, ROS react with nitrogenous bases and deoxyribose, causing significant oxidative reactions, which can cause mutations, oxidised bases and abnormal secondary structures, such as R-loops. We recently showed that increased ROS levels could cause elevated levels of TOP1ccs and this process was dependent on R-loop formation; this was also associated with increased ALT-pathway activity [13].

Crucially, due to the dependence of the ALT-pathway on DNA damage, excessive activation of pathway activity can lead to cell death due to its overwhelming genomic instability, the so-called hyper-ALT phenotype [15]. In this work, we tested that hypothesis that there would be synergy between DPC-forming drugs and ROS-generating treatments in ATRX-deficient cells, and this synergy could be used to sensitise cells to DPC-forming chemotherapy.

## Results

**Figure 1:**
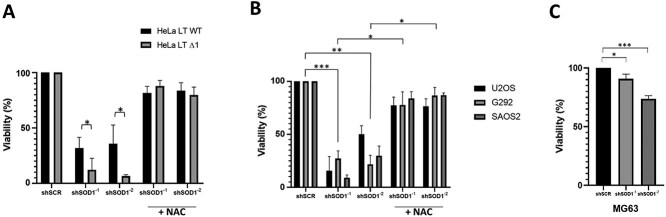
ATRX-deficient cells are highly sensitive to silencing of *SOD1* gene expression. (A) Cell viability of both HeLa LT ATRX-WT and ATRX Δ1 cells was decreased upon silencing of *SOD1*; the effect was significantly greater in the cells lacking ATRX. The effect was ameliorated upon co-treatment with NAC. (B) Testing of a panel of ALT-positive osteosarcoma cell lines showed that cell viability was significantly reduced upon silencing of *SOD1* gene expression and this was largely reversed upon co-treatment with NAC. Significance was tested for average of the 3 cell lines, to give an overall significance of treatment in ALT-positive sarcomas. (C) Testing of MG63, an ALT-negative (telomerase positive) osteosarcoma cell line showed that, whilst cell viability was significantly reduced upon silencing of *SOD1* gene expression, the effect was much milder than that observed in the ALT-positive cell panel. In all panels, cell viability (measured via cell titre Glo) is normalised to shControl (shSCR). Error bars represent standard deviation.

An isogenic pair of HeLa-LT cell lines with either ATRX-wildtype (WT) or ATRX-null were treated with two silencing sequences to *SOD1*, with effective gene knockdown and generation of reactive oxygen species (ROS) previously shown [13]. Cell viability assay was performed, showing that ATRX-null cells had a small, but significant increased sensitivity to silencing of SOD1, as compared to ATRX-WT cells (Fig. 1A). The effect was ameliorated by concurrent treatment with the anti-oxidant N-acetylcysteine (NAC). A panel of well characterised ALT-positive osteosarcoma cell lines was subsequently treated with the same shRNA sequences to *SOD1*. U2OS and SAOS2 are ATRX-null, whilst G292 cells harbour a mutation in *DAXX*, the binding partner of ATRX [16]. This demonstrated that all three cell lines were highly sensitive to *SOD1* silencing, with average cell viability of less than 50% at 5 days (Fig. 1B). The effect was largely reversed by co-treatment with NAC (Fig. 1B). An ALT-negative/telomerase-positive osteosarcoma cell line (MG63) was also tested—this showed that whilst there was some decreased viability in response to silencing of *SOD1*, the effect was much milder than that observed in ALT-positive osteosarcomas (Fig. 1C).

**Figure 2:**
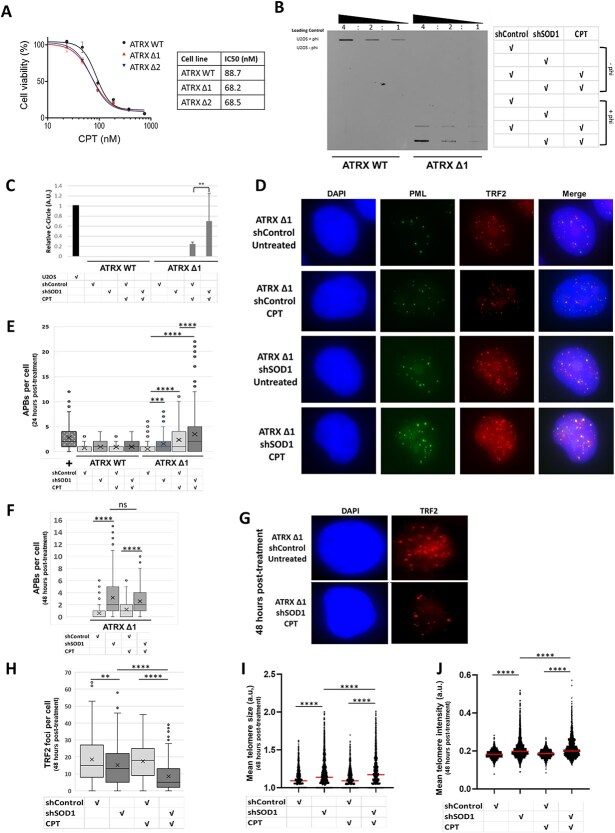
Co-treatment with CPT and shSOD1 has an additive effect on induction of ALT-pathway activity. (A) Cell titre Glo viability assay demonstrated that HeLa-LT cells lacking ATRX had increased sensitivity to camptothecin (CPT). Error bars represent SEM of 3 independent biological repeats, with each biological repeat containing 4 technical repeats. (B and C) At the 24-hour timepoint, C-circle assay showed that co-treatment of cells with both shSOD1 and CPT had a synergistic effect, with increased levels of C-circles beyond the additive amount observed in single treatments; C-circles are a cardinal marker of ALT-pathway activity. Normalised C-circle levels are expressed as arbitrary units relative to the ALT-positive osteosarcoma cell line U2OS, which was used as a positive control on the same membrane; data from 3 independent replicates presented. (D and E) At the 24-hour timepoint, analysis of APBs (another cardinal marker of ALT-pathway activity) confirmed that dual treatment had a complementary effect on cells lacking ATRX. (F) At the 48-h timepoint, the previously observed effect of treatment with shSOD1 and CPT was not observed, with no significant difference between single agent treatments and dual-treated cells. APB number in U2OS cells is included as a positive control (labelled +), to illustrate that the APB numbers reached upon dual treatment are reflective of ALT levels in a natural ALT-positive cell line. (G) The telomeres of dual-treated cells appeared abnormal in both size and number at the 48-hour timepoint. (H-J) At the 48-hour timepoint, there was evidence of severe telomere clustering in cells lacking ATRX treated with both shSOD1 and CPT, as evidenced by a decrease in telomere number (H), an increase in telomere size (I) and increased telomeric intensity (J).

The chemotherapeutic agent camptothecin (CPT) acts to stabilise TOP1ccs, thereby causing replication and transcriptional stress. This drug has previously been shown to increase activity of the ALT pathway [12]. Cell viability assay confirmed that ATRX-deficient cells had significantly increased sensitivity to CPT, as compared to ATRX-WT cells; the estimated CPT IC50 in ATRX-WT cells was 88.72 nM, whereas in *ATRX*-knockout cells, this decreased to 68.2 nM (Δ1) and 68.5 nM (Δ2) (Fig. 2A). Given this sensitivity of *ATRX* knockout alone, the potential synergism of dual treatment with both shSOD1 and CPT was assessed.

Cells were first transduced with shRNA (either shControl or shSOD1), then following successful selection, treated with CPT and analysed for evidence of ALT-pathway activity at 24 h and 48 h post-drug treatment. ALT pathway activity was assessed by two methods, C-circle assay and immunofluorescent imaging of APBs. C-circle assay performed at the 24-hour timepoint demonstrated that dual treatment with shSOD1 and CPT had a marked cumulative effect in ATRX-deficient cells; no induction of C-circles was seen in ATRX-WT cells (Fig. 2B and C). At the 24-hour timepoint, both CPT-treated and shSOD1-treated ATRX-deficient cells showed significant induction of APBs, with a dramatic additive effect of dual treatment (Fig. 2D and E). The level of APBs seen was similar to that observed in the natural ALT-positive osteosarcoma cell line, U2OS.

At the 48-hour timepoint, whilst single CPT-treatment or shSOD1-treatment of ATRX-deficient cells was associated with a strong increase in APBs, it was found that co-treatment (both CPT and shSOD1) did not have further effect (Fig. 2F). This was surprising, given the strong effect observed at the 24-hour timepoint. Further analysis showed that the dual-treated cells appeared very abnormal, with a small number of very large and bright TRF2 foci, suggestive of telomeric clustering or loss (Fig. 2G). The unexpected results regarding APBs might, therefore, be an artifact of this change in telomeric foci number. Further analysis of the cells at the 48-hour timepoint found evidence of significant telomere clustering, with a significant decrease in average telomere number and concurrent significant increase in telomere size and brightness (Fig. 2H-J). Telomere clustering is a sign of induction of the hyper-ALT phenotype, indicating that a cell is in genetic crisis [17–19]. There was no indication of telomere clustering in ATRX-WT cells.

**Figure 3:**
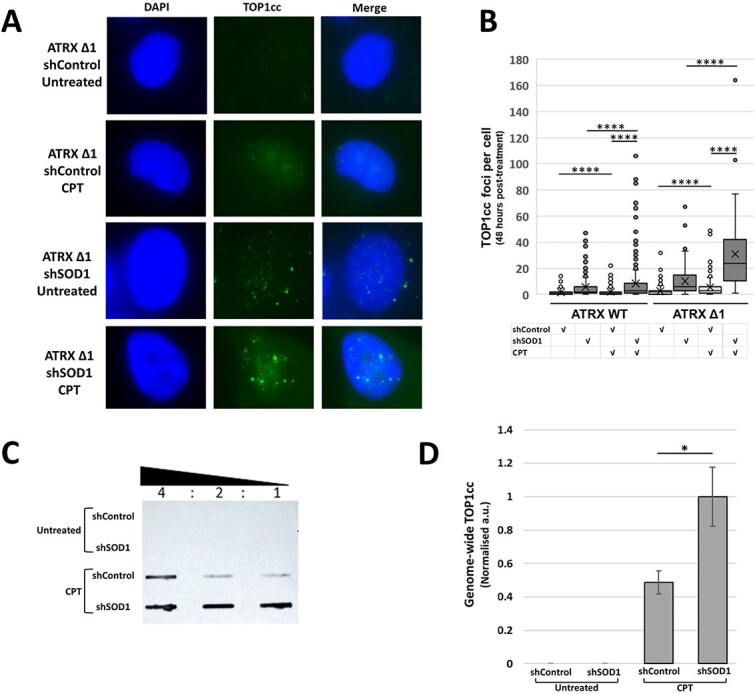
Co-treatment with shSOD1 and CPT causes a marked accumulation of TOP1cc in ATRX-deficient cells. (A and B) representative immunofluorescent images of HeLa LT-ATRX Δ1 cells and quantification showing accumulation of TOP1ccs upon treatment with CPT and/or shSOD1. (C and D) representative blot and quantification of RADAR assay confirmed the finding that dual treatment with CPT and shSOD1 had a synergistic effect on genome-wide levels of TOP1cc in ATRX Δ1 cells—With the total exceeding the additive value observed upon single treatments (data from 2 independent replicates).

CPT acts through stabilisation of TOP1cc, and silencing of *SOD1* gene expression has also been associated with accumulation of TOP1cc [13]. It was considered, therefore, whether there was a synergistic effect between the two treatments on cellular levels of TOP1cc. Firstly, immunofluorescent imaging showed a marked accumulation of TOP1cc with both CPT-treatment or shSOD1-treatment alone; the effect was more marked in ATRX-deficient cells as compared to ATRX-WT cells (Fig. 3A and B). There was a notable cumulative effect of the number of TOP1ccs when the two treatments were combined, but again this was far more striking in ATRX-deficient cells (Fig. 3A and B). RADAR assay was conducted to further verify the results, confirming the significant cumulative effect of combined treatment (Fig. 3C and D).

**Figure 4:**
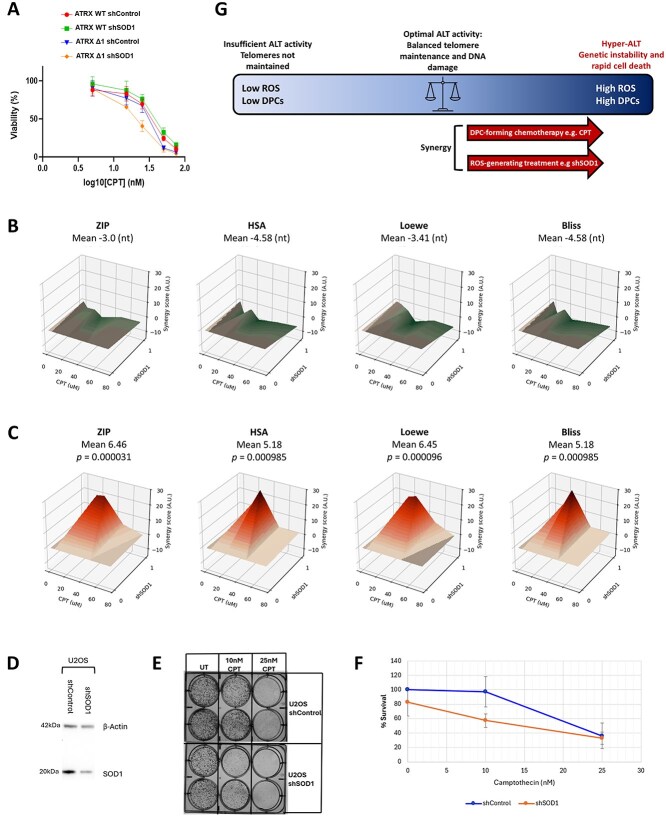
Silencing of SOD1 and camptothecin have a synergistic effect in ATRX-mutant cells. (A) Cell titre glo viability assay showed that pre-treatment with shSOD1 reduced the IC50 of CPT in ATRX-null cells, without affecting ATRX-WT cells (*p* = 0.0084). This confirms the hypothesis that pre-treatment with shSOD1 can be used to sensitise ATRX-deficient cells to CPT chemotherapy. (B) Analysis by four methods demonstrated that there was no significant synergy between shSOD1 and CPT treatment in ATRX-WT cells, with mean value not meeting the threshold of > 5 (non-threshold, nt). (C) Analysis found that there was modest, but significant synergy between shSOD1 and CPT treatment in ATRX-null cells, with mean value meeting the threshold of > 5 across all four tests. (D) Western blot showing successful knockdown of *SOD1* in U2OS cells using shRNA lentiviral transduction. (E) Representative image of a clonogenic survival assay showing that there is an increased effect of CPT when this is combined with *SOD1* knockdown. (F) Quantification of clonogenic survival assay in U2OS cells treated with CPT for 14 days, demonstrating a trend towards significant combinatorial effect (*p* = 0.06 at 10 nM CPT). Error bars represent the standard deviation (SD) across two biological replicates, each with two technical replicates; data normalised to shControl of each biological replicate. (G) Hypothetical framework for the use of hyper-ALT as a novel therapeutic approach in ALT-positive cancer cells.

Given the marked effects of co-treatment with shSOD1 and CPT on TOP1cc accumulation, ALT-pathway activity and induction of genetic crisis, the effect of dual treatment on cell survival was assayed. Cell viability assay was performed at a range of CPT concentrations (0–75 nM), following pre-treatment with either shControl or shSOD1. It is interesting to note that silencing RNA treatment alone—even with control sequence—reduced the IC50 of HeLa LT cells, as compared to the values obtained without pre-treatments (Fig. 2A). However, pre-treatment with shSOD1 did not sensitise ATRX-WT cells to CPT treatment, with no difference in IC50 observed (*p* = 0.2309) (Fig. 4A). There was, however, significantly increased sensitivity to CPT treatment in ATRX-deficient cells pre-treated with shSOD1 (*p* = 0.0084) (Fig. 4A). The IC50 of CPT in *ATRX-*WT control cells was determined as 25.6 nM. The CPT IC50 was significantly lower in dual-treated cells, with a value of 14.68 nM.

Synergy between shSOD1 and CPT treatment was statistically analysed using Bliss, Loewe, HAS and Zip scores [20, 21]. There was no synergy observed between the two treatments in ATRX-WT cells (Fig. 4B). Conversely, some synergy was observed between shSOD1 and CPT treatment in ATRX-null HeLa LT cells, with modest statistical significance (Fig. 4C). Given the limitation of analysing synergy between a binary treatment (presence/absence of silencing RNA), this modest result lends good support to the hypothesis that the synergistic relationship between silencing of SOD1 and CPT treatment could be used to pre-sensitise ATRX-deficient cells to chemotherapy.

Therefore, we went on to test co-treatment with CPT and shSOD1 in a natural ALT-positive, ATRX-negative osteosarcoma cell line, U2OS. Expression of SOD1 protein was greatly reduced following shSOD1 treatment, albeit not completely ablated (Fig. 4D) [13]. We assessed cellular viability using clonogenic assay, as this is a second accurate method for assessing the effects of therapy on cellular survival. This suggested that combination treatment might result in greater cell death than either treatment individually, although statistical significance was borderline (*p* = 0.06) (Fig. 4E and F).

## Discussion

ALT-positive cells exist in a precarious balance—whilst they rely on a threshold level of ALT-pathway activity to maintain telomere length, excessive activation of the pathway causes genetic instability and rapid cell death—the hyper-ALT phenotype [15]. Several approaches have been described which might induce hyper-ALT, including silencing of the Fanconi-related gene, *FANCM*, and stabilisation of G-quadruplexes [17, 22, 23]. Our recent work showed that formation of DPCs, such as TOP1ccs, could initiate the ALT-pathway in ATRX-null cell lines and was likely to be able to increase ALT-activity in ALT-positive cells [12]. Further, high levels of ROS appear to present one way in which DPCs can accumulate in ALT-positive cells, possibly through the formation of R-loops [13]. In this work, we explored the hypothesis that these two approaches could be combined, and in combination would provide a robust method for induction of hyper-ALT in ATRX-deficient cells (Fig. 4G).

The data showed that, as expected, both increasing ROS levels and chemotherapy which could increase DPC levels had the effect of increasing ALT-pathway activity in cells which lacked ATRX. ROS levels were increased through silencing of *SOD1*, an important anti-oxidant gene. CPT was used as a direct TOP1 poison, which acts to stabilise TOP1ccs on DNA. Each of these two treatments was shown to robustly increase C-circles and APBs, two cardinal markers of ALT-pathway activity. Importantly, though, we showed that combining the two therapies—with initial silencing of *SOD1* gene expression, followed by CPT treatment—had a strong additive effect on ALT-pathway activity. At only 24 h after drug treatment, there was a significant cumulative increase in both C-circles and APBs upon dual treatment, as compared to either treatment in isolation. This effect was not observed in ATRX-WT cells. Importantly, we also observed evidence of hyper-ALT in dual-treated ATRX-deficient cells at 48 h post-drug treatment. In the dual-treated cells, we saw significant telomere clustering—as evidenced by massive decrease in telomeric foci number, alongside increased size and intensity. This is strong evidence of genetic instability and hyper-ALT. It is important to note that HeLa LT cells are telomerase-positive and are *not* a natural ALT-positive cell line. These cells were used as it allowed careful investigation of the initiation and level of activity of ALT-pathway in a controlled, near-isogenic system. Based on findings from our prior work, we hypothesised that the combination of DPC formation and increased ROS could lead to the emergence of the ALT phenotype. Our results suggest that the dual treatment drove the cells from a state of no ALT pathway activation to one of increased ALT activity.

In this work, we found that several osteosarcoma lines—such as U2OS, G292 and SAOS2—had varying levels of sensitivity to silencing of SOD1. Further, a clonogenic survival assay demonstrated that there was a trend towards a sensitising effect between shSOD1 and CPT in U2OS cells—although further doses and replicates would need to be investigated before any firm conclusions can be drawn. It would be interesting to explore these questions further, in U2OS and other ALT-positive osteosarcoma lines, as cell-specific difference in anti-oxidant expression might necessitate different approaches in different tumour subtypes. It is possible that silencing of SOD1 is not the optimal choice of targets, and dependency screening of key anti-oxidant genes could reveal new potential targets. A further expansion of the work could consider the effect of pre-treatment with shSOD1, or other redox-targeting shRNA, or other DPC-forming chemotherapies, such as etoposide (TOP2 poison) or olaparib (PARP inhibitor).

Outcomes for many ALT-positive cancers, such as osteosarcoma, high-grade glioma and neuroblastoma remain poor, when compared to non-ALT equivalent cancers [3–5]. This necessitates new therapeutic strategies which complement current chemotherapy regimes. In the UK, DPC-promoting chemotherapies, including camptothecin-derivatives (such as irinotecan) are used in treatment protocols for cancers which are frequently ALT-positive. Osteosarcoma is treated with the protocol defined in the EURAMOS trial, and relies on a backbone of methotrexate, doxorubicin and cisplatin chemotherapies [24]. Cisplatin is known to induce DPCs, due to the drugs ability to form ternary covalent bonds between the DNA and protein, linked by the platinum moiety [25]. Whilst doxorubicin does have some ROS-inducing properties, our data suggests that adding another ROS-inducing treatment (such as shSOD1) might potentiate the DPC-promoting effects of cisplatin, thereby enhancing treatment efficacy. Our data suggests that addition of a ROS-inducing agent to these regimes would be beneficial, due to the observed synergy in ATRX-deficient cells. In our ongoing work, we are expanding on these observations—with a detailed study of relevant ROS-inducing and DPC-promoting agents. This will include rationale combinations of treatments in appropriate cellular systems, which have immediate clinical relevance to current chemotherapy regimes for individuals tumours types. These assays add significant pre-clinical evidence that there is synergy between these two approaches, and potentially pave the way for further development of this novel therapeutic strategy in animal models.

A further consideration is whether ROS-generating pre-treatment approaches could be used to decrease patient exposure to genotoxic chemotherapy agents—could pre-treatments mean that the same effect could be achieved with lower doses of chemotherapy? In some ALT-cancer types (such as low-grade glioma or early-stage tumours), where current chemotherapeutic approaches are often curative and good prognosis is expected, focus is shifting to the risk of adverse events associated with genotoxic chemotherapies, including long-term morbidity, late-effects, and the emergence of secondary cancers—this being particularly relevant to ALT-positive cancers, as the majority occur in children and young people [26]. In those patients who have been stratified as having lower-risk disease by standard clinical scoring systems, it is plausible that pre-treatment with ROS-generating therapies could allow lowered dosage of cytotoxic chemotherapy drugs, such as etoposide and irinotecan (a CPT derivative), thereby decreasing adverse outcomes and long-term risks, without adversely affecting prognosis.

In both cases—either adding to current protocols, or reducing cumulative doses of chemotherapy—consideration must be given to the timing and duration of shSOD1 or any other potent ROS-inducing chemotherapy. The cellular and genetic effects of acute elevation of ROS can be quite different to those observed following chronic exposure, and malignant cells are highly adept at adapting to chronic oxidative stress [27]. In future work, it will be important to assess the optimal timing and duration of potent ROS-inducing therapies, to ensure a balance between promoting DPCs and avoiding chronic oxidative stress related adaptations.

The delivery method for shSOD1, or any other gene-based ROS-generating treatment, must also be investigated. Oncolytic viral therapy is one possible option for silencing RNA delivery. Oncolytic virus therapy is the broad term for any therapy that uses viruses to kill malignant cells and is considered a type of cancer immunotherapy. Successful viruses induce cell death either directly, or indirectly through immune response and preferentially target the cancer cell [28]. One oncolytic virus—T-Vec—is already in routine clinical use for treatment of malignant melanoma, but a large number of other viruses are in phase 1–3 clinical trials for a multitude of cancer types [29, 30]. For example, intertumoral injection of an shRNA-expressing lentivirus has been demonstrated to have therapeutic effects in gastric cancer, and systemic therapy with adenoviruses has had promising early results in several other cancer types [31–33].

Further, ATRX-deficient cells might be uniquely vulnerable to oncolytic viral infection, due to the role of ATRX in early viral responses. ATRX is a key component of PML-nuclear bodies, which control viral infection as part of the cell and nucleus-associated intrinsic antiviral response and, therefore, ATRX-deficient cells might be particularly susceptible to viral infection [34–38]. This means that ATRX-null, malignant cells are infected by oncolytic viruses at doses lower than that which can infect normal cells. One group specifically demonstrated that ATRX-null cancers were highly-sensitive to HSV oncolytic viruses, providing firm evidence that this approach is valid and promising in these tumours [37]. This would be an important way by which unwanted side-effects of silencing of genes could be limited and will be an important aspect of further pre-clinical testing of any such therapies. Other methods for delivery of silencing oligonucleotides which could be considered include chemical modification, bioconjugation and the use of nanocarriers, although these approaches remain largely experimental in cancers at present [39]. It should be noted, however, that antisense oligonucleotide therapies are now routinely used for several inherited neurological conditions, such as spinal muscular atrophy and Duchenne’s Muscular Dystrophy—so although a lot of work still remains to be done, it is not implausible that these therapy-delivery approaches could be used for cancer [40].

Our data contributes to understanding genetic and cellular factors which drive ALT-pathway activity and provides an important proof-of-concept that combining ROS-generating therapies with DPC-forming chemotherapy has a synergistic effect and potentiates the hyper-ALT phenotype. It is likely that such a combination approach would have a synthetic lethal effect in ALT-positive cell lines and future studies will investigate this conjecture. It is hoped that this research will pave the way for future studies in more cellular systems, animal models and eventual clinical trials, with the aim of improving survival and reducing long-term adverse effects on those affected by ALT-positive tumours.

## Materials and methods

### Cell lines and cell culture conditions

Previously generated HeLa LT cells (both ATRX-wildtype and ATRX-null) were used, as described in [12]. HeLa LT cells are a subclone of HeLa (telomerase-positive) that have notably long telomeres and have previously been shown to be amenable to ALT induction [12, 13, 41]. G292, U2OS and SAOS2 (ALT-positive) human cell lines were purchased from ATCC. All cells were cultured at 37°C in 5% CO_2_ and maintained in Dulbecco’s Modified Eagle’s Media (DMEM), which was enriched with 10% foetal calf serum, 1% L-glutamine, and 1% PenStrep (Gibco). To maintain a confluency no higher than 90%, cells were split every 2–4 days using 0.05% trypsin/EDTA (Gibco).

### Treatments of cell with drugs

Prior to ALT status evaluation or TOP1cc quantification, cells were exposed to the genotoxic drug camptothecin (CPT) for 24–48 h at a concentration of 50 nM. Treatment for the RADAR assay consisted of a 10 μM CPT treatment for 1 hour prior to cell lysis. Cell Titre Glo™ assay employed a CPT dosage range of 0–75 nM. Clonogenic survival assay used two doses of CPT at a concentration of 10 nM and 25 nM. Each test included a negative control that used phosphate-buffered saline (PBS) instead of the drug treatment. CPT was purchased from Sigma-Aldrich (PHL89593). Cells were treated with 1.5 mM of NAC concurrently to assess reversal upon anti-oxidant treatment (Sigma-Aldrich, A9165).

### Western blotting

Western blotting was performed using a standard protocol. Superoxide dismutase 1 (SOD1) mouse antibody (Santa Cruz sc-17 767, 1:200), mouse anti-tubulin antibody (Abcam ab4074, 1:50000), and rabbit anti-SOD1 antibody (Cambridge Bioscience A303-812A, 1:500) were the primary antibodies utilised. The secondary antibodies employed were goat anti-rabbit IgG HRP (Thermo Fisher Scientific 31 460, 1:5000) and rabbit anti-mouse IgG HRP (Sigma A9044, 1:5000).

### shRNA knockdowns

To silence *SOD1,* lentivirally packaged shRNA was used; each experiment also included a scrambled control sequence (shControl/shSCR). Lentiviral packaging of the commercially available MISSION shRNA SOD1 (NM_011434) plasmid DNA was performed by the MRC WIMM Viral Genome Facility. To determine the minimum lethal concentration of puromycin for untransduced cells, kill curves were performed at the 48-hour timepoint. Cells were plated in a 6-well format at a density of 300 000 cells per well. The lentiviral mixture consisted of 35 μl of shRNA-packaged lentivirus, 215 μl of DMEM, and 0.3 μl of polybrene. Puromycin selection was initiated 72 h after transduction at a concentration of 3 μg/ml and continued for up to 8 days before cells were either harvested or fixed for subsequent assays. Knockdown efficiency was confirmed by Western blot analysis. A scrambled shRNA sequence (shControl/shSCR) was included as a negative control in all experiments.

### Immunofluorescence

Approximately 50 000 cells were seeded onto circular 13 mm #00 thickness glass coverslips in 24-well plates. Media was aspirated from cells and washed in PBS, pre-permeabilised in 0.5% Triton X-100 (Sigma) in PBS for 1 min on ice. To fix cells onto coverslips they were treated with 4% paraformaldehyde (made up from 16% stock in PBS, Thermo Fisher Scientific) at room temperature for 20 min. After fixation, cells were washed three times with PBS for 5 min each, then permeabilised on ice using 0.5% Triton X-100 for 6 min. Following permeabilisation, cells were washed three additional times with PBS for 5 min per wash, and then blocked for 1 hour in blocking solution (1% BSA in PBS). After blocking, cells were incubated for at least 1 hour with primary antibodies diluted in blocking buffer. Samples were then washed four times with PBS-T (PBS with 0.1% Tween-20) for 5 min each, followed by a 1-hour incubation with fluorescently labelled secondary antibodies, also diluted in blocking buffer. Samples were washed three more times with PBS-T for 5 min each, mounted in VectaShield containing DAPI, and visualised using a DeltaVision widefield microscope. Imaging and analysis were performed as previously described [12]. Each assay was performed with a minimum of two independent biological replicates and analysis of > 150 cells.

### APBs

The primary antibodies used were rabbit anti-TRF2 (Novus Biologicals NB110–57130, 1:500), and mouse anti-PML (Santa Cruz sc-966, 1:300). The secondary antibodies used were goat anti-rabbit Alexa Fluor 568 (Life Technologies A11036, 1:3000), and goat anti-mouse Alexa Fluor 488 (Life Technologies A11029, 1:3000).

### TOP1ccs

The immunofluorescence (IF) protocol was used for TOP1cc IF, with one change—the permeabilization step was extended from 5 to 20 min and the following antibodies: anti-TOP1cc (Merck, MABE1084, 1:300) primary antibody, and goat anti-mouse Alexa Fluor 488 (Life Technologies, A11029, 1:3000), secondary antibody.

### C-circle assay

Pellets of 1 × 10^6^ cells were made for each sample, and genomic DNA was extracted using Thermo Fisher Scientific’s PureLink Genomic DNA Extraction Kit. A 20 μL rolling circle amplification reaction was performed containing 7.5 U of φ29 polymerase (New England Biolabs), 0.1% Tween-20, 200 μg/ml BSA (New England Biolabs), and 1 mM each of dTTP, dGTP, and dATP (all from New England Biolabs), diluted in nuclease-free water. Reactions were incubated in a PCR cycler (Bio-Rad T100 Thermal Cycler) for 8 h at 30°C, followed by a 20-min incubation at 65°C. Each run included a negative control (lacking φ29 polymerase) and a positive control (U2OS genomic DNA).

Amplified samples were then diluted with 180 μl of 2× SSC buffer and applied to a Zeta-Probe membrane (Bio-Rad) using a slot blot filtration manifold (Bio-Rad BIO-DOT, 48-well). The membranes were air dried for 15 min at room temperature and subsequently UV-crosslinked using the ‘Auto Crosslink’ setting on a Stratalinker 2000 (UV-A). The membranes were pre-hybridised with 10 mL of DIG Easy Hyb buffer (Roche) for 20 min at room temperature on a roller. Hybridisation was carried out in a 37°C oven for 2 h using a 3′DIG-labelled [CCCTAA]_5_ probe diluted in 10 mL of DIG Easy Hyb to a final concentration of 40 nM.

Following hybridisation, membranes were washed twice for 5 min each in MS wash buffer (0.1 M maleic acid, 3 M NaCl, 0.3% Tween-20, pH 7.5), and then blocked for 30 min at room temperature in MS blocking buffer (1% milk and 1% BSA in 0.1 M maleic acid, 3 M NaCl, pH 7.5). Anti-DIG-AP Fab fragments (Roche) were added to the blocking buffer at a 1:20000 dilution, and membranes were incubated for 30 min at room temperature.

Membranes were then washed three times for 15 min each with MS wash buffer and incubated with 2 mL of CDP-Star chemiluminescent substrate (Roche) for detection via X-ray film. Analysis of blots was carried out as previously described [12]. All assays were performed using at least two independent DNA samples for each test condition.

### RADAR (rapid approach to DNA adduct recovery) assay

The RADAR assay was performed as described in [13], with minor modifications. Approximately 5 × 10^5^ cells were treated with CPT (as described above) for 1 hour. Following drug treatment, the medium was aspirated, and cells were lysed directly on the plate using 1 ml of DNAzol Reagent (Invitrogen). Nucleic acids were precipitated by adding 0.5× volume of 100% ethanol, incubating at −20°C for 10 min, and centrifuging at maximum speed at 4°C for 15 min. The supernatant was discarded, and the resulting pellet was washed twice with 75% ethanol, followed by centrifugation for 10 min at maximum speed. The nucleic acid pellet was then resuspended in 200 μL of freshly prepared 8 mM NaOH and rotated overnight at 4°C to ensure complete solubilization.

The resulting samples were slot-blotted onto a PVDF membrane and probed using standard Western blotting techniques, as described above. The antibodies used were mouse anti-TOP1 (Santa Cruz, sc-32 736, 1:200) and rabbit anti-mouse IgG HRP (Sigma, A9044, 1:50000).

### CellTitreGlo assay

On 96-well plates, 300 cells were seeded with 90 μL of the cell culture medium. After 6–8 h, an equivalent volume of medium with twice concentration CPT was added, to give the final desired drug concentration range (0–75 nM of CPT). Cell proliferation was evaluated using the CellTiter-Glo 2.0 Reagent (ProMega), as per manufacturer’s instructions. The IC50 value was calculated from dose–response data using curves created in GraphPad Prism v8.0.

### Clonogenic survival assay

U2OS, an ALT-positive cell line, was treated with either shControl or shSOD1 as decribed above. Transfected, puromycin-selected cells were seeded at clonal density (2500 cells per well) in 6-well plates and incubated for 24 h before drug treatment. Cells were then treated with two doses of camptothecin (10 nM or 25 nM) and left for 14 days. An untreated control was included for both shControl U2OS and shSOD1 U2OS. On the final day, media was aspirated, and wells were rinsed with PBS. To fix the cells, 70% ethanol was added to each well and incubated at 4°C for 20 min. Fixed cells were stained with Crystal Violet staining solution (0.5% w/v Crystal Violet in 50% methanol) for 30 min at room temperature. After staining, the solution was aspirated, plates were washed three times with water, and allowed to air dry before colony counting. Once dry, images of the stained 6-well plates were captured using the Invitrogen iBright (ThermoFisher Scientific) with universal settings and analysed using the ImageJ plugin ‘Colony Area.’ Details of this pipeline have been published previously [42]. Two biological replicates were carried out for this assay, each with two technical replicates.

### Quantification and statistical analysis

Social Science Statistics Calculator (https://www.socscistatistics.com/) and GraphPad Prism 9 were utilised for the statistical analysis of the assay findings. For non-parametric unpaired data (such as APBs), the Kruskall-Wallis test was applied. For parametric unpaired data (such as IC50), the Student’s t-test was applied. To examine the strength of the impact of differences between two groups, Cohen’s delta was utilised. This specific notation is used in all figures to indicate significance: ^*^P < 0.05. ^**^P < 0.01, ^***^P < 0.001, ^****^P < 0.0001.

Synergism was assessed using four mathematical models (Zip, HSA, Loewe, Bliss) on SynergyFinder+ [20]. To adjust for the binary nature of shSOD1 treatment (presence/absence), three doses of shSOD1 were computed: shSOD1 = 1 arbitrary units (AU); shControl = 0 AU of shSOD1; shControl = 1×10^−12^AU of shSOD1. This lattermost dummy dataset was comprised of the mean value of each CPT dose in shControl. A significant antagonism is considered to be a mean score of <−5 across all four test scores (with corresponding *p* value < 0.05), whilst significant synergism is considered to be a mean score of > 5 across all four tests [21]. Strong synergism would expect a mean value > 10 across all four tests [20, 21]. Data for shControl and shSOD1 was plotted, excluding the dummy set as follows: surface plots were generated in Python using matplotlib and scipy.interpolate.griddata to visualize interpolated Z-values across a 2D coordinate plane. For each plot, discrete XYZ coordinates were interpolated over a uniform meshgrid using linear interpolation and Z-values mapped to a 3D surface plot using plot_surface from mpl_toolkits.mplot3d.
